# Upper extremity contact pressure measurement in robot-assisted pelvic surgery

**DOI:** 10.1007/s11701-024-01951-5

**Published:** 2024-04-20

**Authors:** Naoki Kimura, Yuta Yamada, Yuji Hakozaki, Jun Kaneko, Jun Kamei, Satoru Taguchi, Yoshiyuki Akiyama, Daisuke Yamada, Tetsuya Fujimura, Haruki Kume

**Affiliations:** 1 Department of Urology, National Center for Global Health and Medicine, Shinjuku-ku, Tokyo, Japan; 2 Department of Urology, Graduate School of Medicine, The University of Tokyo, Tokyo, Japan; 3 Department of Urology, Jichi Medical University, Shimotsuke City, Tochigi Japan

**Keywords:** Robot-assisted pelvic surgery, Contact pressure, Upper extremity complication, Trendelenburg position, Shoulder pain

## Abstract

Upper extremity complications are often a problem in robot-assisted pelvic surgery (RAPS) with the lithotomy-Trendelenburg position (LT-position). This study focused on upper extremity contact pressure (UEP) and examined the relationship between UEP and upper extremity complications. From May 2020 to April 2022 at the University of Tokyo Hospital, UEP was measured in 155 patients undergoing RARP and 20 patients undergoing RARC. A total of 350 sets of UEP were investigated in this study. UEP was measured using a portable interface pressure sensor (Palm Q, Cape CO., Kanagawa, Japan) in the preoperative lithotripsy position (L-position), preoperative LT-position, and postoperative L-position. UEP was increased in the preoperative LT-position than in the preoperative L-position (right side 5.2 mmHg vs. 17.1 mmHg, left side 5.3 mmHg vs. 17.1 mmHg, P < 0.001, respectively), and was decreased in the postoperative L-position than in preoperative LT-position (right side 17.1 mmHg vs. 10.8 mmHg, left side 17.1 mmHg vs. 10.6 mmHg, P < 0.001, respectively). Eleven upper extremities developed shoulder pain. UEP of the preoperative LT-position tended to be higher in the upper extremity exhibiting shoulder pain (25.6 mmHg (15.4–30.3) vs. 17.1 mmHg (12.0–24.4) P = 0.0901). UEP measurements may help prevent postoperative shoulder pain.

**Supplementary Information:**

The online version contains supplementary material available at 10.1007/s11701-024-01951-5.

## Introduction

The steep Trendelenburg position is commonly used for acquiring a better surgical view by moving the abdominal viscera in the direction towards the diaphragm in robot-assisted pelvic surgery (RAPS) [1]. It has been reported that RAPS can be performed safely in patients associated with comorbidities such as cerebrovascular, venous thromboembolism (VTE), and heart disease [2]. However, upper extremity neuropathy (UEN) occurs more frequently in laparoscopic prostatectomy and robot-assisted radical prostatectomy (RARP) than in open radical prostatectomy [3]. UEN may occur in patients associated with the additional weight burden at the shoulder due to the steep Trendelenburg position [4]. Carrying heavy loads can cause paralysis and sensory disturbances in the upper limbs due to vascular insufficiency and nerve plexus tension [5–7]. Accordingly, the long operative time and obesity are risk factors of UEN [8–10]. However, it is unclear regarding the relationship between contact pressure and positioning-related complications in patients undergoing surgeries associated with steep Trendelenburg position. Therefore, this study focuses on the clinical impact and upper extremity pressure (UEP) in patients undergoing RAPS.

## Methods

### Patients’ characteristics and surgical techniques

Two hundred and thirty-four patients underwent RARP for prostate cancer and 32 patients underwent RARC for bladder cancer at the University of Tokyo Hospital from May 2020 to April 2022. All surgeries were performed using the da Vinci surgical robot system (Intuitive Surgical, Sunnyvale, Calif., USA). The number of patients who underwent measurement for contact pressure was 155 in RARP and 20 in RARC. A total of 350 sets of UEP measurements in 175 patients were analyzed for age, body mass index (BMI), surgical time, and console time.

This study was approved by the ‘Ethics Committee of the Tokyo University Hospital’ (# 3124) and was conducted in accordance with the Helsinki Declaration. Written informed consent was obtained from each patient before surgery, including the use of surgical photographs.

### Contact pressure measurements

**Fig. 1:**
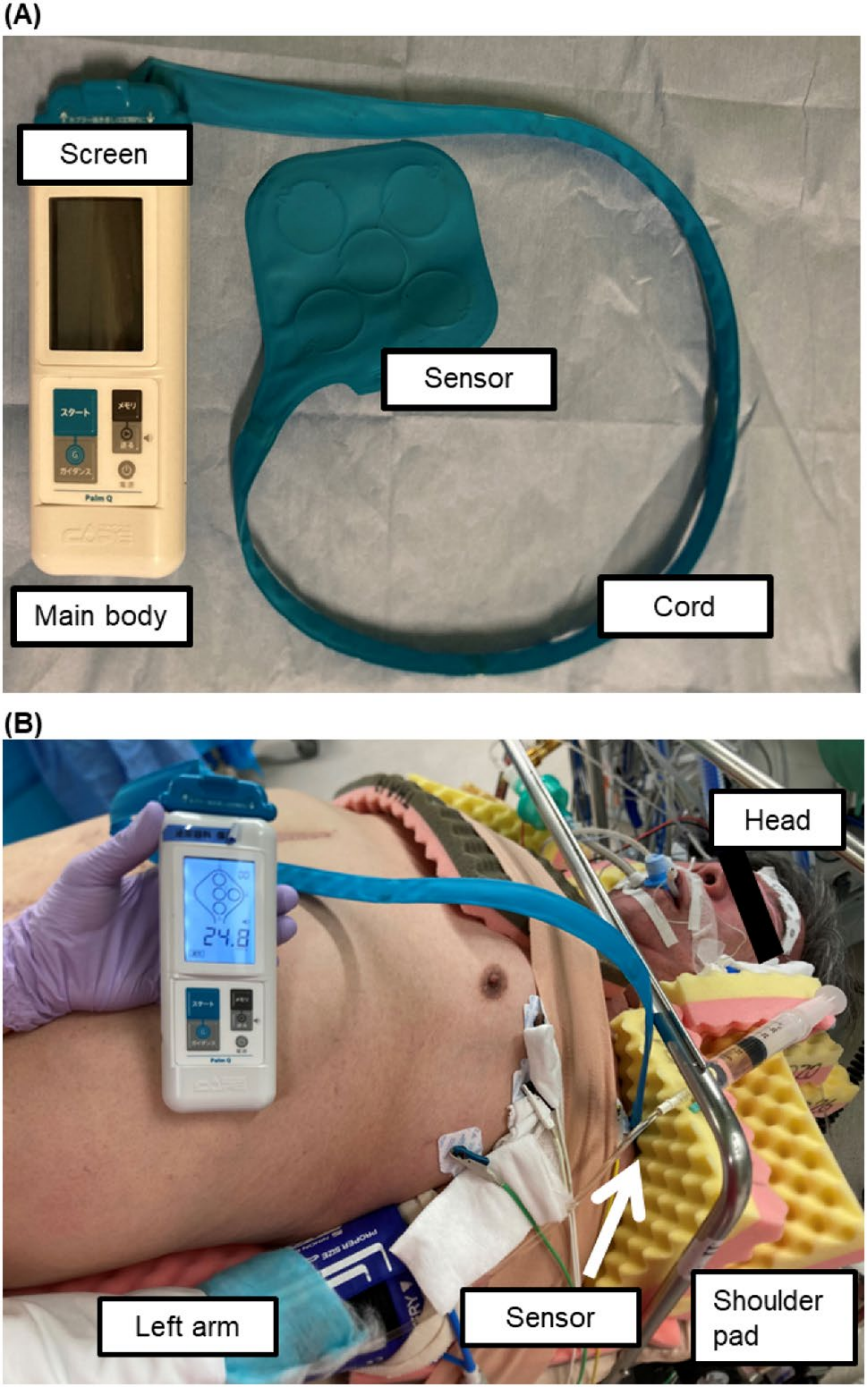
**A** Structure of portable interface pressure sensor (Palm Q, Cape CO., Kanagawa, Japan). It consists of a main body which has a screen and ‘start’ button, cord, and 5 sensors. **B** The picture of measurement of contact pressure. The left shoulder is supported by a cushion pad and Wakasugi side support apparatus (Mizuho Corporation, Tokyo, Japan). The sensor of the device (Palm Q) was placed between the shoulder and cushion pad at the shoulder

Pink pad (Xodus Medical Inc, Pennsylvania, USA) or magic bed (Okada Medical Supply Co., Tokyo, Japan) was laid under the body of the patients. Cushion pads were used between the upper part of the shoulder skin and magic Bed which was secured to the shoulder by a shoulder support apparatus (Mizuho Corporation, Tokyo, Japan). After general anesthesia, all patients were placed in the lithotomy position (L-position). Contact pressure was measured using a portable interface pressure sensor (Palm Q, Cape CO., Kanagawa, Japan) before preoperative disinfection [11] (Fig. 1A). The sensor of the device was placed between the shoulder and cushion pad at the shoulder (Fig. 1B). Patients were placed in a 25-degree Trendelenburg position (lithotomy-Trendelenburg position (LT-position)), and contact pressure was measured again. Postoperative L-position contact pressures were measured after postoperative clean drape removal.

### Statistical analyses

EZR version 1.61 (Jichi Medical University Saitama Medical Center, Saitama, Japan) was used for statistical analysis. Median values for all parameters were rounded to integer values. The correlation between UEP and age, console time, surgical time, BMI, and shoulder pain was assessed by the Wilcoxon rank sum test. A P-value < 0.05 was defined as statistically significant and a P-value < 0.1 was defined as a statistical trend.

## Results

Patients’ characteristics are shown in Table 1. The number of patients that had records of contact pressure measurements in cases with RARP and RARC was 155 and 20, respectively. Across the entire cohort, the median values (IQR) of age, BMI, surgical time, and console time were 71 years old (64–75), 23.5 kg/m^2^ (21.7–25.7), 295 min (221–389), and 229 min (168–311), respectively. One patient complained of unilateral shoulder pain and 5 patients complained of bilateral shoulder pain. The median values (IQR) of the upper extremity pressure (UEP) in preoperative L-position, preoperative LT-position, and postoperative L-position were 5.3 mmHg (1.7–8.9), 17.1 mmHg (12.1–24.8), and 10.6 mmHg (7.3–15.7), respectively (Supplementary Table S1).

**Table 1:** Patient’s characteristics and surgical data

	RARP (N = 155)	RARC (N = 20)	Entire cohort (N = 175)
Age (years), median (IQR)	71 (64–75)	72 (71–82)	71 (64–75)
BMI (kg/m^2^), median (IQR)	23.6 (21.7–25.7)	22.6 (21.0–24.6)	23.5 (21.7–25.7)
Surgical time (min), median (IQR)	280 (215–350)	599 (524–640)	295 (221–389)
Console time (min), median (IQR)	208 (159–274)	509 (411–531)	229 (168–311)
Shoulder pain positive number			
Unilateral	1	0	1
Bilateral	5	0	5
Upper extremity neuropathy	0	0	0

*RARP* robot-assisted radical prostatectomy, *RARC* robot-assisted radical cystectomy, *IQR* interquartile range, *BMI* body mass index

**Fig. 2:**
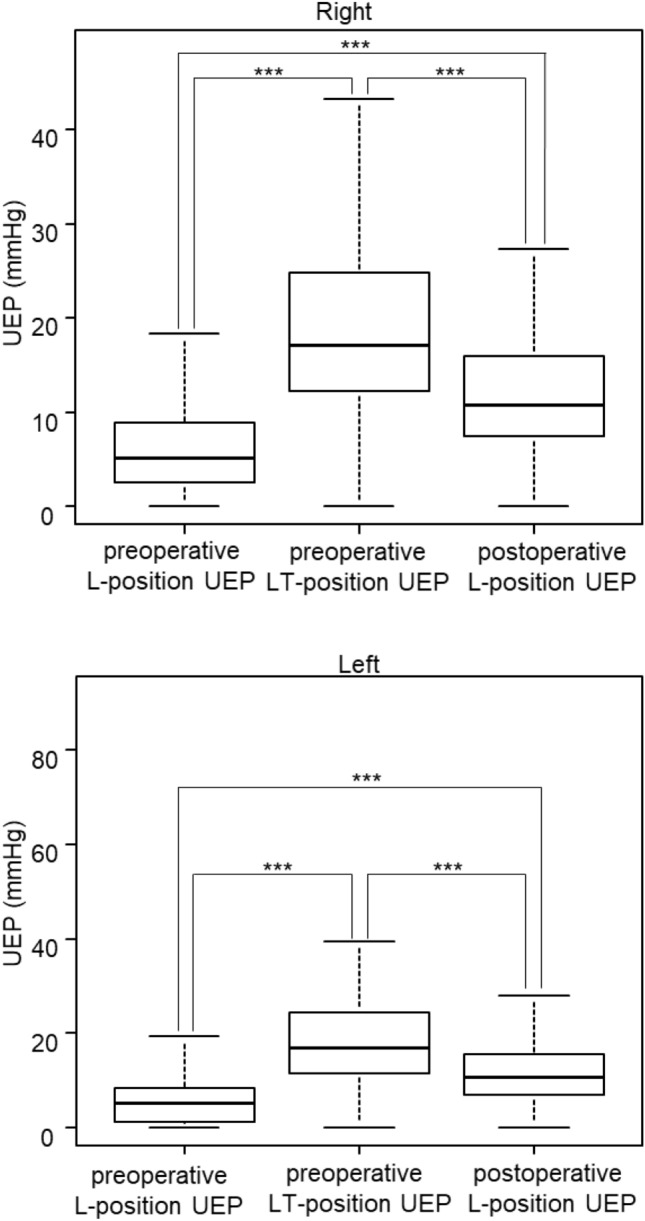
Measurement of upper extremity pressure (UEP). UEP is compared among positioning. Wilcoxon rank sum test. ***: P < 0.001

The relationship between UEP vs. age and UEP vs. BMI was examined. As a result, both age and BMI were not associated with UEP at any positions (Supplementary Fig. S1). The UEP changes at L- and LT-position before and after surgery were investigated. UEP was increased in the preoperative LT-position than in the preoperative L-position (right side 5.2 mmHg vs. 17.1 mmHg, left side 5.3 mmHg vs. 17.1 mmHg, P < 0.001, respectively), and was decreased in the postoperative L-position than in the preoperative LT-position (right side 17.1 mmHg vs. 10.8 mmHg, left side 17.1 mmHg vs. 10.6 mmHg, P < 0.001, respectively) (Fig. 2). No significant relationship was found between UEP elevation and clinical parameters (Supplementary Table S2 and S3).

There were 11 upper extremities associated with shoulder pain. The relationships between shoulder pain and clinical parameters are shown in Table 2. No significant difference was observed between shoulder pain and any clinical parameters. UEP of the preoperative LT-position tended to be higher in the upper extremity exhibiting shoulder pain (25.6 mmHg (15.4–30.3) vs. 17.1 mmHg (12.0–24.4) P = 0.0901) (Table 2).

**Table 2:** The relationship between shoulder pain and other parameters

	Shoulder pain, negative (N = 339)	Shoulder pain, positive (N = 11)	P value
Age (years), median (IQR)	71 (64–75)	68 (62–73)	0.348
BMI (kg/m^2^), median (IQR)	23.5 (21.7–25.5)	23.6 (23.2–25.9)	0.286
Surgical time (min), median (IQR)	298 (221–396)	281 (196–311)	0.124
Console time (min), median (IQR)	230 (168–312)	215 (155–274)	0.599
Preoperative L-position UEP (mmHg)	5.3 (1.3–8.7)	5.1 (4.3–12.0)	0.312
Preoperative LT-position UEP (mmHg)	17.1 (12.0–24.4)	25.6 (15.4–30.3)	0.0901
Postoperative L-position UEP (mmHg)	10.8 (7.3–15.8)	9.4 (7.4–14.3)	0.427

*IQR* interquartile range, *BMI* body mass index, *UEP* upper extremity pressure, *L-position* lithotomy position, *LT-position* lithotomy-Trendelenburg position

## Discussion

This study examined the changes in UEP in RAPS and evaluated the relationship between UEP and shoulder pain. UEP increased in the Trendelenburg position, and high UEP tended to produce postoperative shoulder pain.

The force of gravity moves the patient's body toward the cephalic direction when the operating table is tilted. On the other hand, frictional forces are simultaneously generated to keep the patient’s body from moving. The UEP reflects the repulsive force from the shoulder pad towards the caudal direction when the operating table is tilted to the Trendelenburg position. Theoretically, these forces are balanced to maintain the object's position. However, the increase in UEP due to changes in the patient’s body position is likely related to complex factors. For instance, it may be related to the increased force on the shoulder resulting from body fluid movement, subcutaneous emphysema, and the patient's body shift.

The contact pressure is calculated as P = F / A (P: pressure, F: force, A: area) [12]. In the Trendelenburg position, body fluid moves from the legs toward the heart by gravitational displacement due to body inversion [13]. In addition, the RARP procedure can cause subcutaneous emphysema around the neck [14]. Moreover, the cephalad move of the patient occurs due to the Trendelenburg position in RAPS [15]. On the other hand, when the patient is in the Trendelenburg position, the changes in the mean contact area for the shoulders did not show a significant difference [16]. When considering the effects of body fluid movement, subcutaneous emphysema, and the patient’s body shift, these factors increase the force on the shoulder. However, the contact area of the shoulder remains unchanged. Consequently, a change in the patient’s body positioning may increase UEP.

In the present study, shoulder pain was observed in 3.5% of all cases and is considered a relatively common complication; high UEP in the LT position may be a useful marker to predict complications of the upper extremity in RAPS. With regards to the contact pressure in the lower extremity, reports including previous literature define the safe range of the contact pressure as less than 32 mmHg based on capillary pressure [11, 12]. However, the present study showed that the median contact pressure of patients associated with shoulder pain was 25.6 mmHg (15.4–30.3). It seems that the safe range of the contact pressure in the upper extremity needs to be investigated in further studies.

While this study was successful in identifying the relationship between shoulder contact pressure and postoperative shoulder pain, there is one limitation that should be noted. That is, the mechanism of postoperative shoulder pain is composed of complex factors. For example, a heavy load on the shoulder can cause shoulder injury and pain [17]. Subacromial impingement syndrome, caused by heavy physical loads and shoulder shape, is another common complaint of shoulder pain [18]. Additionally, in laparoscopic surgery, hyperextension of the diaphragm due to pneumoperitoneal pressure can cause postoperative shoulder pain [19].

## Supplementary Information

Below is the link to the electronic supplementary material.

Supplementary file1 (DOCX 57 KB)

## Data Availability

The datasets used for the current study are not publicly available since ongoing clinical studies based on the same database are in progress, but they can be used by a reasonable request to the corresponding author.
