# Supplementary material for: Upper extremity contact pressure measurement in robot-assisted pelvic surgery
Source: J Robot Surg. 2024 Apr 20;18(1):179. doi: 10.1007/s11701-024-01951-5 (PMC11032272; doi:10.1007/s11701-024-01951-5)
Supplement: Supplementary file 1 — Supplementary file1 (DOCX 57 KB) [file 11701_2024_1951_MOESM1_ESM.docx]

**
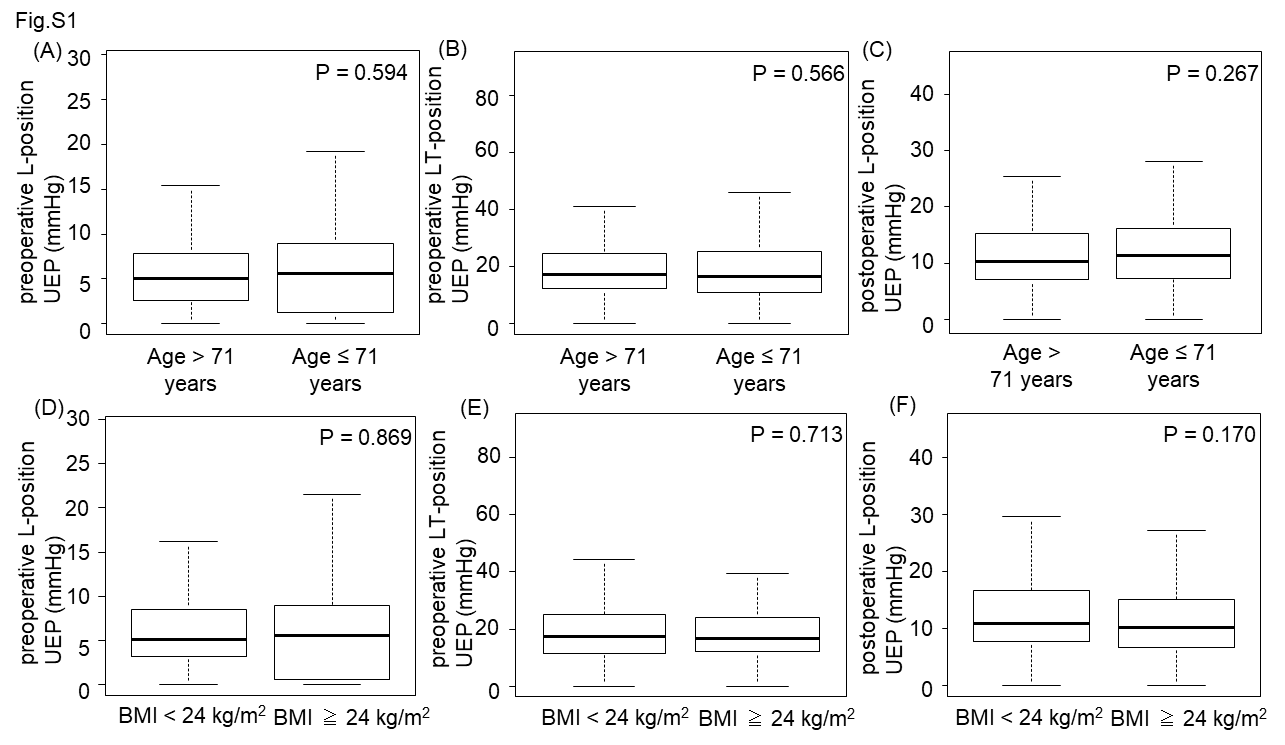
Supplementary Fig.S1.** Relationship between UEP vs. age and UEP vs. BMI. Wilcoxon rank sum test. **(A-C)** The relationships between age and **(A)** preoperative L-position UEP, **(B)** preoperative LT-position UEP, **(C)** preoperative LT-position UEP. **(D-F)** The relationships between BMI and **(D)** preoperative L-position UEP, **(E)** preoperative LT-position UEP, **(F)** preoperative LT-position UEP.

Supplementary Table S1 Data of upper extremity pressure (UEP) (N = 350)

| Variable | Median (IQR) |
| --- | --- |
| Preoperative L-position UEP (mmHg) | 5.3 (1.7-8.9) |
| Preoperative LT-position UEP (mmHg) | 17.1 (12.1-24.8) |
| Postoperative L-position UEP (mmHg) | 10.6 (7.3-15.7) |

IQR: interquartile range, L-position: lithotomy position, UEP: upper extremity pressure, LT-position: lithotomy-Trendelenburg position

Supplementary Table S2 Relationship between ΔUEP (preoperative LT-position – preoperative L-position) elevation and clinical parameters

| Variable | Elevation of contact pressure | | | | | |
| --- | --- | --- | --- | --- | --- | --- |
|  | Right upper extremity | | | Left upper extremity | | |
|  | Not elevated | Elevated | P value | Not elevated | Elevated | P value |
| Age (years) |  |  |  |  |  |  |
| < 71 | 5 | 76 | 0.885 | 3 | 77 | 0.835 |
| ≥ 71 | 4 | 85 |  | 5 | 84 |  |
| BMI (kg/m^2^) |  |  |  |  |  |  |
| < 24 | 7 | 90 | 0.345 | 4 | 93 | 0.946 |
| ≥ 24 | 2 | 71 |  | 4 | 68 |  |
| Pain (numbers) |  |  |  |  |  |  |
| Negative | 9 | 157 | 1.0 | 8 | 156 | 1.0 |
| Positive | 0 | 4 |  | 0 | 5 |  |
| Console time (minutes) |  |  |  |  |  |  |
| < 230 | 4 | 82 | 0.941 | 3 | 82 | 0.679 |
| ≥ 230 | 5 | 77 |  | 5 | 77 |  |

BMI: body mass index, UEP: upper extremity pressure, L-position: lithotomy position, LT-position: lithotomy-Trendelenburg position

Supplementary Table S3 Relationship between ΔUEP (postoperative L-position – preoperative L-position) elevation and clinical parameters

| Variable | | | | Elevation of contact pressure | | | |
| --- | --- | --- | --- | --- | --- | --- | --- |
|  | Right upper extremity | | | | Left upper extremity | | |
|  | Not elevated | Elevated | P value | | Not elevated | Elevated | P value |
| Age (years) |  |  |  | |  |  |  |
| < 71 | 14 | 63 | 0.726 | | 14 | 62 | 0.677 |
| ≥ 71 | 19 | 69 |  | | 13 | 75 |  |
| BMI (kg/m2) |  |  |  | |  |  |  |
| < 24 | 17 | 80 | 0.452 | | 13 | 83 | 0.325 |
| ≥ 24 | 16 | 52 |  | | 14 | 54 |  |
| Pain (numbers) |  |  |  | |  |  |  |
| Negative | 31 | 130 | 0.376 | | 26 | 133 | 1.0 |
| Positive | 2 | 2 |  | | 1 | 4 |  |
| Console time (minutes) |  |  |  | |  |  |  |
| < 230 | 16 | 65 | 1.0 | | 13 | 67 | 1.0 |
| ≥ 230 | 17 | 65 |  | | 13 | 69 |  |

BMI: body mass index, UEP: upper extremity pressure, L-position: lithotomy position, LT-position: lithotomy-Trendelenburg position
